# Health Behaviors and Psychological Distress Among Conscripts of the Lithuanian Military Service: A Nationally Representative Cross-Sectional Study

**DOI:** 10.3390/ijerph17030783

**Published:** 2020-01-27

**Authors:** Brigita Mieziene, Arunas Emeljanovas, Vida Janina Cesnaitiene, Daiva Vizbaraite, Renata Zumbakyte-Sermuksniene

**Affiliations:** 1 Lithuanian Sports University, 44221 Kaunas, Lithuania; arunas.emeljanovas@lsu.lt (A.E.); vida.cesnaitiene@lsu.lt (V.J.C.); daiva.vizbaraite@lsu.lt (D.V.); 2 Lithuanian University of Health Sciences, 44307 Kaunas, Lithuania; renukz@gmail.com

**Keywords:** conscripts, physical activity, nutrition, smoking, alcohol consumption

## Abstract

The decline in healthy behavior in young people is a concern for public health in general and for country’s defense. The aim of this study is to identify and compare health behaviors and psychological distress between male conscripts enlisted and rejected for military service. This cross-sectional study included 1243 men aged 19–26 years (mean age 22.50 ± 2.43 years). We assessed health behaviors (physical activity, adherence to healthy eating patterns, cigarette smoking, and alcohol consumption) and psychological distress. Among all conscripts, 44.7% were physically inactive, 50.2% had low adherence to healthy nutrition, 9.6% were heavy drinkers, 62.3% were current smokers, and 9.1% had high psychological distress level. Compared with physically inactive conscripts, physically active conscripts were more likely to be enlisted (adjusted odds ratio (OR) = 1.42; 95% confidence interval (CI) 1.11–2.03). Compared with current nonsmokers, current smokers were less likely to be enlisted (OR = 0.58; CI 0.39–0.86). Compared with conscripts with a high distress level, those with a low distress level were almost four times more likely to be enlisted (OR = 0.26; 95% CI 0.12–0.55). Adherence to guidelines for healthy eating and alcohol consumption was not significantly related to enlistment. These findings suggest that health behaviors in male conscripts are unsatisfactory. That is, about half are physically inactive, have a poor diet, and smoke, and nearly one in 10 is a heavy drinker and has a high psychological distress level. The enlisted conscripts were more likely to be sufficiently physically active and less likely to be a current smoker or have a high distress level. Early intervention programs to provide a heathier population of young men for conscription should focus on mental well-being and target health-related behaviors such as physical activity and not smoking. Preferably, these should be implemented as health education programs in schools to help prevent the development of adverse health behaviors among young men. Governmental policies and strategies are required to enable intersectional collaboration and shared responsibility among the education, military and health sectors.

## 1. Introduction

The decline in youth’s healthy behaviors and related consequences [1] is of concern for public health in general and for national defense in particular, which requires a sufficient number of physically fit and mentally healthy military personnel. In Lithuania, there is evidence that young people do not partake in adequate physical activity; that is, only around 30% of students aged 18 years comply with the recommendation to be active ≥1 h on at least 5 days a week [2]. The trends for health-related physical fitness among adolescents have deteriorated in the past 20 years, and the indicators of cardiorespiratory fitness have decreased by nearly 50% during this period [3]. Only 13–14% of high school students comply with recommendations for healthy nutrition [4,5], 11% of young people consume alcohol [6]. Moreover, 22% experience psychological distress [7]. Multiple health behaviors, psychological distress, and their associated risk factors, such as obesity and poor physical fitness, are associated with poor mental health outcomes, the risk of cardiovascular disease, type 2 diabetes, certain types of cancer [8,9], depression [10,11], and anxiety [12]. Unhealthy behaviors also impose an economic burden; for example, one study estimated that physical inactivity alone costs USD 53.8 billion in 2013 for healthcare worldwide [13].

Health is the main criterion for accepting or rejecting young men into military service (MS) and is strongly related to the ability to perform military duties. Unsatisfactory results of youth recruitment to Lithuanian MS have been presented: on average, 58.6% of Lithuanian young men proceed through the full procedure of military enlistment. Among those who do not pass these procedures, 33%–37% experience psychological problems, 29%–33% have cardiovascular diseases, and 13% have musculoskeletal problems [14]. Other countries face similar problems of rejection from MS. For instance, analysis of the reasons for rejection from MS in the USA found that about 22% of rejections were because of problems with bones or joints, flat feet, or hernias, 15% because of organ defects, 13% because of defects of the cardiovascular system, 12% because of nervous system or mental problems, and 10% because of communicable diseases [15]. In the USA, Hispanic men appear to have a better health profile than their white and black peers, except for the prevalence of overweight, which is higher in Hispanic men [16].

Although health behaviors alone are not criteria for enlistment into MS, they might explain the reasons for some instances of rejection. Given that some health behaviors are risk factors for the occurrence of many lifestyle-related diseases [17], it is critical to identify whether and how health behaviors differ between young men who are deemed eligible and those who are ineligible for MS and to take appropriate actions to prevent adverse health behaviors from their onset.

Soldiers must be both physically and mentally healthy. However, mental health issues are among the main factors for rejection from MS [14,15]. Identifying psychological distress along with health behaviors might help to provide a more complete understanding of health indicators in conscripts as psychological distress is an indicator of mental health [18].

Given the associations between many health-related behaviors, the complex analysis of a set of risk behaviors instead of evaluation of individual associations may help to reduce the risk of missing potential confounders for enlistment into MS [19].

Several studies have examined psychological distress, health behaviors [20,21], and changes in health behavior [22] during MS. Enlistment is based on a medical examination of draftees, which may reject unhealthy individuals. As a result, these studies have evaluated health behaviors in relatively healthy youth but have not examined whether health behavior is related to the rejection of military recruits.

The aim of this study is to identify and compare health behaviors and psychological distress between male conscripts rejected for and enlisted into MS. We expect to find that healthier behaviors and low distress level would be related to a higher rate of enlistment into MS.

## 2. Materials and Methods

### 2.1. Study Design and Procedure

This nationally representative cross-sectional study was performed among Lithuanian conscripts. In Lithuania, 9-month-long MS is compulsory. In accordance with the conscription procedure of the Lithuanian Armed Forces, a list of potential draftees is created by an automatic electronic selection system each year and includes male Lithuanian citizens of compulsory MS-eligible age (19–26 years). Other male and female young adults can express their wish to be conscripted on a priority basis.

A stratified random sampling was used. There are four centers for military recruitment in Lithuania that recruit conscripts during the whole year. The data were gathered in all four centers for 4 months from June to October 2018. Each conscript in these centers was approached during this period and asked to sign a consent form to participate in the study and to complete the study questionnaire. There was an equal chance (probability) that participants included in the study would be enlisted or rejected for MS.

The decision to accept a recruit for enlistment into MS is made by medical experts and is based on an individual medical examination and previous medical reports. The criteria for rejection from MS are defined in Order V-1142/V-1139, which provides a list of disorders along with their severity, and is signed by the Ministers of State Defense and Health Care. These criteria also indicate the minimum height requirement: 160 and 155 cm for men and women, respectively. Obesity without comorbid illness is not a reason for rejection from MS [23]. Enlistment in and rejection from MS were identified from the medical records for each study participant.

### 2.2. Participants

In 2018, there was a list of 10,340 conscripts, 209 of them female. This study included 1427 conscripts (which represented 13.80% of the total population), of whom 1296 returned their completed questionnaire along with their consent to participate in the study. The response rate was 90.82%. Female recruits were invited to participate in the study, but because only 53 agreed to participate, they were later excluded because of the small sample size. Finally, 1243 young male potential conscripts were included in the analysis. The participants were aged 19–26 years and their mean age was 22.50 ± 2.43 years.

The Ministry of National Defense of the Republic of Lithuania approved the research. Ethics approval (No SMTEK-28, 2018) was obtained from the Ethics Committee of Lithuanian Sports University. The investigations were carried out following the rules of the Declaration of Helsinki of 1975, revised in 2013. Participants were informed of the tasks in the study before data collection, and all participants gave their informed consent for inclusion before they participated in the study.

### 2.3. Measurements

#### 2.3.1. Physical Activity

The World Health Organization (WHO) defines moderate physical activity as activity that noticeably accelerates the heart rate and includes activities equivalent in intensity to brisk walking or bicycling. Vigorous physical activity causes rapid breathing and substantially increases heart rate, and includes activities such as jogging, aerobic dance, and bicycling uphill [24]. To assess physical activity in the participants in this study, we used the 2005 US Department of Defense Survey of Health Related Behaviors Among Active Duty Military Personnel [21]. The study participants were asked, “During the past 7 days, for leisure-time physical activity, how often did you usually do each of the following?”. Participants were also asked, “During the past 7 days, when you did leisure-time physical activity, how long did you usually do each of the following?” For both questions, detailed descriptions and examples of what constitutes moderate and vigorous physical activity were presented.

In this study, we assessed physical activity during the preceding 7 days instead of the 30 days in the original questionnaire. Also, instead of using categorical answers (such as “5 or 6 days” and “at least 20 minutes”) for each question, we provided the opportunity to write the exact numbers of days, hours, and/or minutes per day. The number of minutes spent in was totaled. Participants whose MVPA was <2.5 h/week were coded as not meeting health-related physical activity requirements and those whose MVPA was ≥2.5 h/week were coded as meeting health-related physical activity requirements [25].

#### 2.3.2. Eating Patterns

A healthy eating pattern was defined as a diet based on whole or minimally processed foods and that included health-protective ingredients and lacked unhealthy items such as fast food and sugar-sweetened beverages [26]. Adherence to a healthy eating pattern was evaluated using the Mediterranean Diet Adherence Screener (MEDAS) [25], which was previously validated in Spanish and German adult populations [26,27]. The MEDAS includes 14 items: two indicate food-intake habits such as the use of olive oil and preference for white versus red meat, and the other 12 items capture the frequency of consumption of olive oil, animal fat, vegetables and fruits, fish, nuts, commercial pastries, sugar-sweetened beverages, and dishes with homemade sauce. Each item was scored as 0 (does not meet the healthy eating criteria) or 1 (meets the healthy eating criteria). The total score was calculated by summing all item scores. The MEDAS score was classified into three categories: ≤7 indicated low adherence, 8–9 indicated medium adherence, and ≥10 indicated high adherence to the Mediterranean diet [26].

#### 2.3.3. Alcohol Consumption

Alcohol consumption was evaluated according to the 2008 US Department of Defense Survey of Health Related Behaviors Among Active Duty Military Personnel [20]. Participants were asked to indicate the number of drinking occasions during the past month with the question, “During the past 30 days, on how many days did you drink alcohol?”. Participants had to identify the quantity of drinks per typical drinking occasion in the table, where columns presented the type of beverage classified as the number of beers (bottle or can = 330 mL), the number of wines (glass = 125 mL), or the quantity of whiskey/vodka (mL), liquor (mL), and other drinks (mL) by indicating the quantity or amount of each of the drinks in the columns. The number of occasions per month when ≥5 drinks were consumed was determined with the question, “During the past 30 days, on how many days did you have 5 or more drinks of beer, wine, or liquor on the same occasion?” (By “drink,” we mean a bottle or can of beer, a wine cooler or a glass of wine, a shot of liquor, or a mixed drink or cocktail. By “occasion,” we mean at the same time or within a couple of hours of each other.) Following the 2008 Department of Defense Survey of Health-Related Behaviors Among Active Duty Military Personnel [20] paper, participants were allocated into three groups according to alcohol consumption: abstinent (0 alcoholic drinks in the past month), light–moderate drinker (1–4 drinks per typical drinking occasion or ≥5 drinks per typical drinking occasion 1–3 times/month), heavy drinker (≥5 drinks per typical drinking occasion ≥1/week (on average) in the 30 days before the survey). The classification was adapted from Mulford and Miller (1960) [28].

To provide a continuous variable indicating the total amount of alcohol consumed, the total number of alcohol units per month was calculated as one standard unit equal to 10 g ethanol. In accordance with the WHO guidelines, the following formula was used: volume (mL) multiplied by the percentage of alcohol and divided by 1000 [29]. 

#### 2.3.4. Cigarette Smoking

Cigarette smoking was evaluated as indicated in the 2008 US Department of Defense Survey of Health Related Behaviors Among Active Duty Military Personnel [20]. Participants were asked to indicate the number of cigarettes smoked in the past month. Given that there is no safe amount of tobacco use, as reported by the 2014 Surgeon General’s Report [30], two categories of smoking were created: current nonsmoker (smoked 0 cigarettes in the past 30 days and never smokers) and current smoker (smoked ≥1 cigarette in the past 30 days).

#### 2.3.5. Psychological Distress

Psychological distress was assessed using the six-item Kessler scale [31]. Participants were asked to evaluate their nervousness, hopelessness, anxiety, restlessness or fidgety feelings, worthlessness, and depression. Each item was scored from 0 (none of the time) to 4 (all the time). The total score was calculated by summing the scores for each item and ranged from 0 to 24 points, with a lower score indicating a lower level of psychological distress. The internal consistency of the scale was good (Cronbach α = 0.876). The summed score was dichotomized as low psychological distress (0–12 points) and high psychological distress (≥13 points) [31].

### 2.4. Covariates

Body mass index (BMI) was evaluated using the self-reported height and weight and was calculated as weight (kg)/height (m^2^). Family income was evaluated by asking participants to rate their current family financial situation as having insufficient income, having average income, having higher than average income, wealthy, and rich. Age (in years) was also used as a covariate.

### 2.5. Statistical Analysis

The data were analyzed using SPSS (ver. 24, IBM SPSS Statistics; IBM Corp., Armonk, NY, USA). Categorical variables are presented as frequencies and percentages. Differences in categorical variables were tested using the chi-squared test. The relationships between independent continuous variables were examined using Pearson correlational analysis because all scaled variables had a normal distribution. Logistic regression analysis was used to identify significant associations between dependent (enlistment into MS) and independent variables (covariates, health behaviors, and psychological distress level) by adding independent variables individually into the next model and producing odds ratios (ORs) and 95% confidence intervals (95% CIs). The final model contained adjusted OR (95% CI). Statistical significance was set at *p* < 0.05.

## 3. Results

Among 1243 conscripts who went through the enlistment procedures, 624 (50.2%) were rejected for and 619 (49.8%) were enlisted into MS (Table 1). Around half were adequately physically active. Among youth enlisted to MS, there were 63.1% of physically active versus 47.3% of physically active among youth rejected for MS (p < 0.01). The level of adherence to the Mediterranean diet was low among half of Lithuanian male conscripts, a little bit less than half of them had medium adherence, and only a small percentage (3.5%) of male conscripts complied with healthy eating patterns. However, there were no significant differences between rejected and enlisted youth (*p* > 0.05). Almost a quarter of all conscripts reported their alcohol abstinence for the previous 30 days, two-thirds reported a light–moderate drinking pattern, and almost one out of ten (9.6%) reported heavy drinking. The percentage distribution after Bonferroni correction indicated that among youth enlisted to MS, there were 6.8% of heavy drinkers versus 12.3% of heavy drinkers among rejected youth (*p* < 0.01). Percentages of light–moderate drinking and abstinence for the 30 previous days were not significantly different (*p* > 0.05) between rejected and enlisted youth. Almost two-thirds of male conscripts were current smokers; however, smoking was more frequent among those who were rejected for MS (67.7%) than among those who were enlisted (57.0%) (*p* < 0.0001). A high level of distress was reported in 9.1% of the total population of male conscripts. However, among rejected youth, high distress had 14.7% of prevalence versus 3.4% of high distress prevalence among enlisted youth (*p* < 0.0001).

Continuous variables for health behaviors and distress were included in the correlational analysis to examine the relationships between the main predictors of enlistment, and separate analyses were performed for the rejected and enlisted conscripts (Table 2). In both groups, there were significant relationships between better adherence to healthy eating patterns and lower smoking levels (r = −0.011 and r = −018 for rejected and enlisted, respectively) and lower psychological distress levels (r = −0.12 and r = −0.22 for rejected and enlisted, respectively). Smoking and distress were also significantly correlated (r = 0.12 in both groups), which showed that a higher psychological distress level was associated with a higher average number of cigarettes smoked per day. In both groups, higher alcohol consumption was related to higher smoking levels (r = 0.22 and r = 0.24 for rejected and enlisted, respectively) and higher distress levels (r = 0.21 and r = 0.27 for rejected and enlisted, respectively). Among the men enlisted into MS, there were also significant associations between greater alcohol consumption and lower adherence to healthy eating (r = −0.12), and between higher compliance to healthy eating patterns and a higher physical activity level (r = 0.13) (*p*_s_ < 0.05).

Enlistment into MS was predicted using multiple logistic regression analysis (Table 3). When entered individually into the model, enlistment into military service was associated with MVPA (OR = 1.97; CI 1.57–2.47), adversely associated with heavy drinking (OR = 0.60; CI 0.45–0.79), current smoking (OR = 0.61; CI 0.49–0.78), and psychological distress (OR = 0.20; CI 0.13-0.33). After controlling for all variables included in the regression analysis in Model 7, the significant predictors of enlistment remained higher MVPA, current non-smoking, and low level of distress. Adherence to healthy eating was not significantly related to enlistment neither entered individually nor after controlling for covariates and other study variables (*p* > 0.05). Among the covariates, only older age predicted a lower chance of being enlisted (OR = 0.77; 95% CI 0.71–0.83). Neither BMI nor perceived family income was associated with enlistment (*p* > 0.05).

## 4. Discussion

This study was aimed at determining the prevalence of healthy behaviors and psychological distress in recruits for MS and at identifying differences in these indicators between young men rejected for and enlisted into MS. The results partly support the premise that young men who are enlisted into MS are more likely to adhere to healthy behaviors and have lower psychological distress levels compared with those rejected for MS.

### 4.1. Physical Activity

In the regression analysis, after controlling for covariates and other study variables, meeting the recommendations for MVPA was significantly associated with being enlisted into MS. Previous studies have shown that physical activity can help prevent 26 different chronic diseases [17] and is related to a lower incidence of metabolic syndrome, lower rates of stress, and better mental health and cognitive functions [17,32,33], all of which are important to being able to serve in the military. Physical activity is also a strong predictor of physical fitness [34], which is a key indicator of physical health during MS. Other research has suggested that physical activity dampens the inflammatory processes in the body [34]. Higher-intensity physical activity confers greater health benefits, such as reduced body fat and central adiposity, and higher cardiovascular fitness in youth, compared with lower-intensity activity [35]. In the current study, 55.3% of all conscripts had an adequate level of MVPA, but this percentage was lower in those rejected for MS (47.4%) than in those enlisted (63.1%) into MS. These results are similar to data reported in the general population of similar age in the Eurobarometer (2017) data, which showed that 55% of male youth in Lithuania and 57% of male youth from other European countries aged 15–24 years engage in physical activity regularly or with some regularity [36]. The physical activity level generally increases in those enlisted into MS after they start their duty because MS enforces its own standards for health behaviors such as physical activity by, for example, requiring daily physical activities. Surprisingly, however, it has been reported that 13% of those serving in the US active duty military perform insufficient exercise [37].

### 4.2. Cigarette Smoking

Along with physical activity, current smoking also predicted enlistment into MS. The logistic regression analysis after controlling for covariates and other study variables indicated that current smokers had a 72% lower chance of being enlisted than nonsmokers. In total, around two-thirds of Lithuanian male youth called to serve in the military are current smokers. However, among those enlisted to the military, there are 10.7% less of them than among those rejected for MS. A study in Taiwan reported that 50.8% of young men in their MS smoked before service [38]; this percentage is 10 percentage points lower than in Lithuania. In the US, 23% of active duty personnel smoke [36], which is much lower than the 57% smoking rate in Lithuanian conscripts enlisted into MS in our study. The statistics for the general Lithuanian population confirm the high prevalence of smoking: one in three men smoke every day in Lithuania. This is the fourth highest rate in the European Union [39]. Smoking is related to obesity [40] and hypertension [41], and is the most relevant risk factor for the burden of disease and mortality [39,42]. A low physical activity level and smoking are the most prevalent health risk factors [43] and were also negative predictors of enlistment for MS in our study.

### 4.3. Psychological Distress

Psychological distress was another significant predictor of enlistment into MS in this study. We found by 11% higher prevalence of high psychological distress among those rejected for than among those enlisted into MS. This effect was independent of health-related behaviors such as physical activity, nutrition, cigarette smoking, and alcohol consumption, which might be interrelated to psychological distress given that these behaviors can act as coping strategies [7,44,45]. However, in total, only 9.1% of these male conscripts reported a high psychological distress level. Another study of the Lithuanian high school male student population, from which the military draws its enlistees, reported a 12.5% prevalence of psychological distress measured using the same Kessler scale [7]. A study in Massachusetts in the USA found that 20% of high school students of both sexes reported depressive symptoms [46]. An Australian study of university students reported a prevalence of high psychological distress of 11.1%–22.5% [44]. A prevalence of high psychological distress of 11%–18% was reported for active duty personnel in the US military, and the prevalence was higher among heavy drinkers than among those who consumed less alcohol [20]. This finding suggests an interdependence of heavy alcohol consumption and psychological distress. This association was identified in the correlational analysis in our study; in particular, of the associations of psychological distress with other health behaviors, the association with alcohol consumption was the strongest.

### 4.4. Alcohol Consumption and Nutrition

Alcohol was consumed by 75.8% of the included youth, and 9.6% were heavy drinkers. These figures are similar to the heavy or risky drinking behavior noted in about 12% of Swiss conscripts [19] and 7.8% of US active duty personnel [36]. In a report on healthy lifestyle factors among adult Lithuanians, the Lithuanian Department of Statistics reported that 34% of the population consumes alcoholic beverages at least once a month [47].

The adherence rates for healthy nutrition among potential conscripts in our study are problematic because only 3.5% fully complied and 46.3% partly complied with the healthy eating recommendations. The Country Health Profiles (2019) reported that 32% of deaths in Lithuania could be attributed to dietary risk factors, a rate that is higher than the 18% in other European countries. Dietary risk has the highest risk for mortality among other health behaviors: smoking is related to 15% and 17%, alcohol consumption to 10% and 6%, and physical inactivity to 5% and 3% of deaths in Lithuania and Europe, respectively [39].

The regression analysis did not find any associations between enlistment and alcohol consumption or dietary habits in the final model after controlling for all study variables included, although the differences in the percentages were shown significant for alcohol consumption. Significantly more of the recruits rejected for MS (12.3%) were heavy drinkers than those enlisted (6.8%). When entered individually into logistic regression, heavy drinking lowered the chances of being enlisted into MS. However, after the addition of covariates and other study variables, this relationship lost its significance. Moreover, study participants are young men and their history of alcohol consumption may be too short to have a noticeable direct effect on their health. This could also be said about their dietary habits. However, other research has shown that both alcohol use and nutrition over the long term are associated with health outcomes such as the risk of diabetes, cardiovascular disease, Parkinson’s disease, certain types of cancer, myocardial infarction, and high blood cholesterol level [48,49,50,51].

Overall, our results correspond with those of other studies where health-related behaviors and psychological distress are linked to physical comorbidities [52], which were the main reasons for rejection from MS. Public health policies are targeted at strengthening health in the total population and its subpopulations, such as young people. Similarly, state authorities are also interested in having physically fit and healthy conscripts, who are mostly young men. Improving health among youth is most effective when started early in life during behavior development and when behavioral changes are easier to implement. Health education in an organized way that targets the young population may be best implemented at school, which provides the best setting for delivering public health interventions at a young age. In particular, physical development of school children could be based on physical literacy that addresses attributes, characteristics, skills, and behaviors that are related to the ability for, and commitment to, a healthy, active lifestyle [53]. From the military perspective, these interventions could also be modeled to capture physical capacity (e.g., physical endurance) and psychological resilience (e.g., resistance to stress) and to emphasize the importance of avoiding substance use or abuse. The development of a healthy future military could start at a young age and involve the objectives of both public health and state defense. To implement this, governmental policies and strategies are required to enable intersectional collaboration and shared responsibility among the education, military and health sectors as well as to set up coordinating mechanisms among sectors. Funding and financial incentives should be provided for intervention programs and implementers. National regulations are needed to enable school administrative and staff to expand their competencies and skills in physical and mental health promotion on a basis of compulsory professional training.

### 4.5. Study Limitations

The study has some limitations. We used a cross-sectional design, and no causal inferences can be drawn. It remains unclear whether the poor health behaviors were the causes or the consequences of the health outcomes, which were the reasons for rejection from MS. Longitudinal studies are needed to evaluate the direction of, and interactions between, the associations demonstrated here. Another limitation is that we did not include objective measures of physical and mental health. Physical and/or mental health may be confounding variables that both prevent conscripts from serving in the military and increase the likelihood of exhibiting unhealthy behaviors and having a high psychological distress level. Another possible confounding factor may be the interaction between psychological distress and health behaviors because the latter can result from the former. However, the logistic regression analysis allowed us to control for this possible confounding effect. That is, the health behaviors that were significant for enlistment remained significant even after psychological distress levels were included in Model 7. Another limitation is that we did not measure the onset of health-damaging behavior, which may have served as a control variable when examining the predictors of enlistment, especially for addictive behaviors such as smoking and alcohol consumption. In addition, we did not assess the use of other substances such as “party drugs,” and we did not obtain a full picture of these conscripts’ use patterns.

Our study also has some strengths. This was a nationally representative sample that provides a picture of the health behaviors and prevalence of psychological distress among young men called to serve in the military and the differences between those who were enlisted and rejected for MS. Further research should investigate the relationships between health behaviors in conscripts and the reasons they are rejected.

## 5. Conclusions

Health behaviors in male conscripts are unsatisfactory because about half are physically inactive, have a poor diet, or smoke, and almost one out of 10 is a heavy drinker and has a high psychological distress level. The latter is a concern because of its association with adverse health behaviors. The enlisted conscripts were more likely to be sufficiently physically active and less likely to be current smokers or have a high psychological distress level. Early intervention programs to provide a heathier population of young men for conscription should focus on mental well-being and target health-related behaviors such as physical activity and smoking. Preferably, these should be implemented as health education programs in schools to help prevent the development of adverse health behaviors among young men. Governmental policies and strategies are required to enable intersectional collaboration and shared responsibility among the education, military and health sectors.

## Figures and Tables

**Table 1:** Descriptive statistics and comparison (chi-squared test) between recruits rejected for and enlisted into military service.

	Total (%)	Rejected for Military Service (%)	Enlisted Into Military Service (%)	χ^2^; df; *p*
MVPA	*N = 1219*			31.0; 1; <0.0001
Low (<2.5 h/week)	44.7	52.6	**36.9**	
Adequate (≥2.5 h/week)	55.3	47.4	**63.1**	
Mediterranean diet	*N = 630*			2.3; 2; 0.315
Low adherence	50.2	51.5	49.4	
Medium adherence	46.3	44.9	48.0	
High adherence	3.5	3.6	2.6	
Alcohol consumption	*N* = 1082			9.2; 2; 0.010
Abstinent (≥30 days)	24.2	23.4	25.0	
Light–moderate drinker	66.2	64.3	68.1	
Heavy drinker	9.6	12.3	6.8 *	
Cigarette smoking	*N = 1179*			16.1; 1; <0.0001
Current nonsmoker	37.7	32.3	**43.0**	
Current smoker	62.3	67.7	**57.0**	
Psychological distress	*N = 1159*			44.4; 1; <0.0001
Low	90.9	85.3	**96.6**	
High	9.1	14.7	**3.4**	

Note: Boldface indicates significant values (*p* < 0.05); * adjusted *p* values for 2 × 3 comparisons using the Bonferroni method. df, degrees of freedom; MVPA, moderate to vigorous physical activity.

**Table 2:** Correlations between the predictors of enlistment for recruits rejected for (above the diagonal) and enlisted into (below the diagonal) military service.

	MVPA (h/Week)	Mediterranean Diet	Alcohol (Units/Month)	Cigarettes (Number/Day)	Psychological Distress
MVPA (h/week)	–	–0.02	0.01	0.05	–0.07
Mediterranean diet	**0.13**	–	–0.06	**–0.11**	**–0.12**
Alcohol (units/month)	–0.03	**–0.12**	–	**0.22**	**0.21**
Cigarettes (number/day)	–0.01	**–0.18**	**0.24**	–	**0.12**
Psychological distress	–0.07	**–0.22**	**0.27**	**0.12**	–

Note: Boldface indicates significant values (*p* < 0.05). MVPA, moderate to vigorous physical activity.

**Table 3:** Multiple logistic regression predicting enlistment to military service from health behaviors and distress in Lithuanian male youth, controlling for family income, body mass index, and age (crude and adjusted odds ratios (ORs)).

	Model 1	Model 2	Model 3	Model 4	Model 5	Model 6	Model 7
Predictors	Odds ratio (95% CI)						Adjusted odds ratio (95% CI)
Family income	1.15 (0.93–1.43)						1.09 (0.87–1.36)
BMI	0.99 (0.95–1.03)						0.99 (0.95–1.03)
Age	**0.76 (0.71–0.82)**						**0.77 (0.71–0.83)**
MVPA (≥2.5 h/week)		**1.97 (1.57–2.47)**					**1.42 (1.11–2.03)**
Mediterranean diet							
Low adherence			Ref.				Ref.
Medium adherence			1.27 (0.96–1.74)				0.90 (0.63–1.29)
High adherence			0.81 (0.34–1.93)				0.83 (0.29–2.39)
Alcohol consumption							
Abstinent (≥30 days)				Ref.			Ref.
Light–moderate drinker				0.52 (0.82–1.49)			1.15 (0.73–1.83)
Heavy drinker				**0.60 (0.45–0.79)**			0.64 (0.31–1.34)
Cigarette smoking							
Current nonsmoker					Ref.		Ref.
Current smoker					**0.61 (0.49–0.78)**		**0.58 (0.39–0.86)**
Psychological distress (high)						**0.20 (0.13-0.33)**	**0.26 (0.12–0.55)**
Nagelkerke R^2^	0.06	0.04	0.01	0.02	0.02	0.06	0.16

Note: Boldface indicates significant values (*p* < 0.05). CI, confidence interval; BMI, body mass index; MVPA, moderate to vigorous physical activity; Ref., reference group.
